# Evaluation of Solvents Used as Keepers in the Determination of Organic Pollutants by GC/MS

**DOI:** 10.3390/molecules25194419

**Published:** 2020-09-25

**Authors:** Łukasz Dąbrowski

**Affiliations:** Department of Food Analysis and Environmental Protection, Faculty of Chemical Technology and Engineering, UTP University of Science and Technology, 3 Seminaryjna Street, 85-326 Bydgoszcz, Poland; lukas@utp.edu.pl; Tel.: +48-523-749-014

**Keywords:** keeper, solvent evaporation, sample preparation, organic pollutants, GC/MS

## Abstract

Solvent evaporation is often used in the sample preparation procedure for the determination of organic pollutants such as polycyclic aromatic hydrocarbons (PAHs), polychlorinated biphenyls (PCBs) and organic pesticides. Because of the loss of analyte during this step, a high-boiling solvent, i.e., a keeper, is often added to the extract before evaporation. However, there are almost no basic studies found in the literature on the selection of keepers for the appropriate type of analytes (keepers are usually selected only on the basis of information provided by various recommendations). In this work, the effect of several keepers (isooctane, toluene, nonane, octanol, dodecane) on the recovery of various analytes (PAHs, PCBs, organic pesticides) was evaluated (during evaporation in a stream of nitrogen, at 40 °C). The analysis of the results obtained for the tested compounds shows that 1-octanol is a universal keeper for compounds with low volatility, i.e., PCBs (average recovery: 97.6%), organochlorine pesticides (average recovery: 95.0%), organophosphorus pesticides (OPPs; average recovery: 99.7%) and higher mass PAHs (average recovery: 91.9%). The use of isooctane as a keeper yields high recoveries for PAHs, regardless of their volatility (average recovery: 95.5%). When using 1-octanol or dodecane as a keeper, the reversed solvent effect (during GC analysis) was noted in relation to volatile analytes causing the distortion of their peaks. Additionally, the phenomenon of loss of some analytes (e.g., OPPs) was observed during evaporation without heating the vials. However, in the case of PCBs, organochlorine pesticides (OCPs) and *o*-hydroxybiphenyl, evaporation under such conditions yields recoveries greater than or equal to 90.0%. The results presented in this work can help in finding a suitable keeper for a specific group of analytes or an alternative to the commonly used one, especially in the case of recovery problems.

## 1. Introduction

Solvent evaporation during the environmental, food and biological sample preparation procedure is a common task used in solvent exchange or to reduce the volume of the extract. Although many miniaturized sample preparation techniques are popular, such as low density miniaturized homogenous liquid–liquid extraction (LDMHLLE) [1], single-drop microextraction (SDME) [2], dispersive liquid–liquid microextraction (DLLME) [3] and others [4], applications that require a "traditional" approach to the determination of polycyclic aromatic hydrocarbons (PAHs), polychlorinated biphenyls (PCBs) and organic pesticides, with solvent evaporation, are still in use. Among several others, evaporation in a stream of nitrogen remains the most popular method when reducing a small volume of solvent or used as a second step in the evaporation procedure [5]. However, this stage is limited by the loss of analytes, especially volatile ones. A well-known method to overcome this problem is to add a keeper before evaporating the solvent. A keeper is usually a high-boiling-point liquid added to the extract in a small volume (usually 20–300 µL and above). According to the review paper on the use of keepers in the analysis of environmental samples published in 2016 [5], there are only a few works with basic studies on the application of keepers, i.e., for polycyclic aromatic hydrocarbons and their derivatives [6,7].

Recently, several new research papers describing analytical procedures with the solvent evaporation step and the use of a keeper have been published. The determination of organic pollutants in samples of various types has been described in studies considering PAHs in contaminated soil samples [8] and particulate matter [9]; chlorpyrifos and other pesticides in river water and sediments [10]; pyrethroid metabolites in human urine [11]; organochlorine pesticides (OCPs) and PCBs in human serum [12,13,14]; PCBs and other persistent organic pollutants (POPs) in fish [15]; and PCBs in waste, leachate and aerosols [16]. In these and other works, the keeper was selected arbitrarily on the basis of previous works, recommendations, standards and regulations [5]. Nevertheless, there is a lack of basic research comparing different keepers (except [6,7]) and considering the influence of heating the sample during evaporation on analyte recovery. Similarly, information on this type of study cannot be found in the literature in relation to a wide range of compounds differing in physicochemical properties.

The aim of this work is the comparison of the effectiveness of decreasing the loss of analytes for various keepers (isooctane, toluene, nonane, 1-octanol, dodecane) during the evaporation of the solvent (dichloromethane) in the stream of nitrogen. After evaporation, the samples were analyzed by GC/MS system. The research included determination of recovery for various analytes (PCB, PAHs, OCPs, organophosphorus pesticides (OPPs) and others) after the evaporation of the solvent in the thermostated vials. Additionally, the influence of conducting this process without heating was also tested. The recovery of the analytes served as a measure of the effectiveness of the keeper in the solvent evaporation process.

## 2. Results and Discussion

All GC/MS chromatograms were integrated, and the peak area of each analyte was used for calculations, also taking into account the area of the internal standard (IS) peak. Calibration curves were determined separately for each compound in dichloromethane (DCM) and in different DCM/keeper mixtures. The determination coefficients (R^2^) for these curves were greater than or equal to 0.99. The practical range of the analyte recoveries for the evaporation process is from 25% (0.156 µg/mL) to 100% (0.625 µg/mL), and only this range was quantified. The results below and above the range of calibration curves were evaluated by simple extrapolation. Additionally, the results were checked for outliers using the Grubbs test.

For more volatile keepers such as isooctane and toluene (Table S1), the solvent evaporation end-point is difficult to determine, and the final volume will vary between evaporations. From a practical point of view, it is much easier to find the evaporation end-point (the point at which the keeper is the only solvent left in the vial) when a less volatile keeper is used. Even if the sample is kept longer than necessary in the evaporator, this does not significantly affect the volatilization of the keeper. Changes in the final volume of the keeper influence the concentration of analytes, and it is necessary to use an internal standard to evaluate these differences. Nevertheless, this correction method can be used when there are not too many differences in the final volume of the keeper.

The time required for complete evaporation (to dry) or evaporation to the volume of the keeper was from 12 to 16 minutes for samples thermostated at 40 °C. For a process without compensation for heat loss (vials in ambient temperature), the evaporation time doubled (to 32–36 min) and the temperature in the evaporation vial dropped by more than 30 °C below the initial temperature to below 0 °C. Due to the longer evaporation process, nitrogen consumption was also about twice as high.

The results of these experiments are shown in Table 1, Table 2, Table 3, Table 4 and Table 5. Recovery of evaporation process together with RSD is presented for all compounds. Additionally, the number of compounds (cpds) with the recovery between 90% and 110% is also given. To simplify the evaluation of results, the average recovery and RSD values were calculated for all compounds. Recoveries above 100% can be explained as a result of random error, and they can also come from impurities present in the keeper.

### 2.1. Influence of the Keeper on Recovery of PCBs

PCBs are a group of substances with low vapor pressure (low volatility) and high logP (high adsorption on the glass surface (Table S2)). The results of the experiments are shown in Table 1. It is easy to see that evaporation of the solvent at 40 °C without (*w*/*o*) adding the keeper (Experiment 1) gives low recoveries, especially for compounds with a lower boiling point. Interestingly, the evaporation process without heat loss compensation (evaporation without heating, Experiment 2) gives very good results: a recovery of about 90% and an RSD usually below 3%.

According to a previously published review paper [5], nonane and dodecane are the most commonly used keepers in the PCB determination procedure. In the latest works describing analytical procedures (for PCBs) using keepers, nonane was used by Sprague M. et al. (2015) [15] and tetradecane was used by Arp H.P.H. et al. (2020) [16]. In the experiments presented in this work, the average recovery for nonane (used as a keeper) was 85.6%, while the average recovery for dodecane was over 80.1%. The highest recoveries were obtained using 1-octanol (with an average RSD = 2.1%). No literature reports have been found regarding the use of 1-octanol for this purpose. PCBs are sometimes analyzed together with compounds with similar properties, such as polychlorinated dibenzo-p-dioxins (PCDDs), polychlorinated dibenzofurans (PCDFs) and polybrominated diphenyl ethers (PBDEs) [15,17]—it can be assumed that the results presented in this work can be also extrapolated to a broader group of substances.

### 2.2. Influence of the Keeper on Recovery of OCPs

The results obtained for OCPs (Table 2) are quite similar to those for PCBs. The highest recoveries were obtained using 1-octanol as a keeper, but evaporation without its addition at ambient temperature (Experiment 2) also gave acceptable results. For nonane, recoveries of about 90% were obtained with low RSDs. When toluene was used as a keeper, the recovery of lower mass OCPs was only improved (compared to Experiment 1), while the recovery of the remaining compounds was relatively low.

In recently published studies on the determination of OCPs (and PCBs) in human blood, Dufour P. et al., used nonane as a keeper in their studies published in 2018 and 2020 [13,14], and Stubleski J. et al. (2018) used tetradecane [12]. This information is partly in line with the results presented in this paper, although the recoveries for dodecane (similar to tetradecane) were not very high (about 80%).

### 2.3. Influence of the Keeper on Recovery of PAHs

In PAH determination by means of GC, there are two solvents usually used as keepers, i.e., toluene and dodecane, but there are also several more that have been mentioned recently: isooctane [8], nonane [9] and other [5]. PAHs are a group of substances with different volatilities, i.e., compounds with a lower molecular weight are more volatile (naphthalene, methylnaphthalenes, acenaphthylene, acenaphthene). This is the main reason the recoveries of these substances are lower in almost all cases (Table 3) except for those using isooctane and nonane as keepers. The loss of these compounds during evaporation is well known in the literature [5,6]. A certain impact on the lower PAH recovery may be due to the fact that they are thermosensitive and photosensitive compounds [18,19].

Evaporation without the addition of a keeper gives very low recoveries for almost all analytes (the average recovery was above 60%). Lower recovery levels for heavier PAHs can be associated with their relatively high logP values (strong adsorption on the glass surface of the vial). The highest recoveries for all compounds were obtained using isooctane as a keeper. In the case of 1-octanol (RT = 6.15–6.65 min) for the five early eluting analytes, the so-called reversed solvent effect can be observed. They elute before or on the peak of the keeper on the chromatogram and are distorted in a way that does not allow their quantification. For other compounds, average recovery levels are high (almost 92%). This is in line with previously published work in which 1-octanol and 1-hexanol were tested as keepers (recovery of over 90% for most of the analyzed compounds) with the final determination by HPLC method [6]. In the case of using dodecane (RT = 6.40–6.70 min) as a keeper, a very weak signal from naphthalene can be seen due to the coelution of these two compounds, of which the keeper has a very high concentration. The mass spectrum of naphthalene is not specific enough (ions 128 and 127 are also present in the dodecane spectrum) to be able to be separated from dodecane.

### 2.4. Influence of the Keeper on Recovery of OPPs and Other Substances

The OPPs are a group of labile substances with moderate logP values and low volatility (Table S5); they can easily be oxidized. There are only a few works that describe the use of keepers in the OPP determination procedure. For example, hexane was used as an added substance to prevent sample evaporation [10] and degradation in water samples [5].

Evaporation of OPPs without the addition of a keeper at 40 °C (Experiment 1, Table 4) gives a recovery of above 60%, while evaporation at ambient temperature causes the peaks of analytes to disappear from the chromatograms (a similar effect can be observed for o-hydroxybiphenyl (Table 5)). This phenomenon can be explained by the adsorption of these compounds on the glass surface of the evaporation vial, which has already been described in the literature [20,21]. In these studies, 1-octanol, nonane and isooctane used as keepers gave the highest OCP recoveries. In the case of pyrimethanil, 1-octanol was also the best keeper used in the solvent evaporation procedure. Analyzing the data for o-hydroxybiphenyl, it can easily be seen that the recovery was above 95% when using nonane and isooctane as keepers. Due to the short retention time (6.85 min), this substance also suffers from the reversed solvent effect caused by 1-octanol and dodecane (similar to early eluting PAHs). Moreover, the mass spectra of o-hydroxybiphenyl and 1-octanol are quite similar, and the analyte cannot be distinguished from keeper by mass spectrum.

## 3. Materials and Methods

### 3.1. Materials and Reagents

Dichloromethane of pesticide residue grade was obtained from Merck (Poznań, Poland). The solvents isooctane, toluene, nonane, 1-octanol and dodecane (purity of at least 99.9%) were purchased from Sigma-Aldrich (Poznań, Poland) and used as keepers. Stock solutions of mixtures of pesticides, PCBs and PAHs were obtained from Supelco (Poznań, Poland). Standard solutions were diluted from the stock solutions with dichloromethane and mixed together. The concentration of each component in the standard stock solution used in the experiments was 1 µg/mL. The names of the compounds in the standard solution are given in Table 1, Table 2, Table 3, Table 4 and Table 5 (and in Tables S2–S5). A solution of triphenyl phosphate (TPH, from Merck) in DCM at the concentration of 60 µg/mL was used as the internal standard (IS). Calibration curves for each analyte in DCM and in different DCM/keeper mixtures were determined separately in the range of 0.156 to 0.625 µg/mL.

### 3.2. Evaporation of Standard Solutions

To evaluate the effect of addition of the keeper on analyte recovery after solvent evaporation, several types of experiments were performed. Each type of experiment was repeated five times. In each case, the standard solution (in DCM) and 100 µL of one of the keepers (in the experiment with the addition of a keeper) were added to a 7 mL vial. Then, the appropriate volume of DCM was added up to a total volume of 6 mL. The concentration of the analytes in a vial was 0.033 µg/mL. Dichloromethane was chosen as the main solvent because it dissolves different types of analytes; therefore, it is very often used in the analysis of OCPs, PCBs, PAHs and many other compounds [5].

Evaporation was carried out using a six-position homemade nitrogen evaporator with a glass water bath and adjustable nozzle position (Figure S1). Webcams were installed in front of the vials to facilitate observation of the evaporation end-point. Nitrogen was introduced onto the surface of the dichloromethane solution through a gas flow manifold terminated with nozzles located about 2 cm above the liquid position. The position of the nozzles was adjusted during the evaporation process to keep approximately the same distance from the liquid surface (at the end of the evaporation process, the distance was greater).

#### 3.2.1. Evaporation without the Addition of a Keeper

Two different types of experiments were carried out: evaporation using a vial thermostat (water bath) operating at 40 °C (Experiment 1) and evaporation without heating, i.e., evaporation vials kept at ambient temperature (Experiment 2). Because this process is endothermic, without compensating for heat loss, this leads to a considerable drop in temperature in the vial. Evaporation was carried out until dry: each vial was individually controlled and removed from the evaporator as soon as the solvent evaporated. The residue was reconstituted in 300 µL of DCM and 20 µL of the internal standard solution in DCM (added to the evaporation vial with the 25 µL HPLC syringe). The vial was shaken vigorously for ~1 min to dissolve the analytes, and then the mixture was transferred to 340 µL autosampler vials. The addition of IS to the evaporation vial allowed the final concentration to be evaluated independently of the final volume—even when the final solution was not quantitively transferred. The sample was analyzed using the GC/MS system.

#### 3.2.2. Evaporation with the Addition of a Keeper

A keeper (100 µL of isooctane, toluene, nonane, 1-octanol, or dodecane) was added to the evaporation vial before the evaporation process. This keeper volume was selected after analyzing the data presented in the aforementioned review article [5] and in recent articles [8,9,10,11,12,13,14,15,16] (100 µL was the most frequently used volume). The volume of the keeper added was also adjusted to the size of the vial (7 mL) in which the evaporation was taking place. All experiments were performed with the thermostat of the vials operating at 40 °C (the experiments were marked with the numbers from 3 to 7). Evaporation was performed until the keeper was the only solvent left in the vial (i.e., approximately 100 µL). Then, 200 µL of DCM and 20 µL of IS solution (in a similar manner as described in Section 3.2.1) were added to the vial. The next steps were carried out as described in Section 3.2.1.

### 3.3. GC/MS Analysis

An Agilent 7890B gas chromatograph equipped with a 7693 autosampler and a 5977B mass-selective detector (Agilent, Santa Clara, CA, USA) was used for the final analysis. The chromatographic column used was an Agilent fused silica capillary column HP-5MS, 30 m × 0.25 mm × 0.25 µm. Helium was used as carrier gas at 1.5 mL/min. The split–splitless injector was operated in pulsed pressure splitless mode with the following program: initial pressure 0.2 MPa (30 psi) for 1.3 min, decreased to constant flow. The purge valve was opened after 1.5 min and the injection volume was 5 µL. The other GC parameters were as follows: injector temperature 290 °C; transfer line temperature 280 °C; oven temperature initially 50 °C for 1.5 min, then ramped to 180 °C at 35 °C/min, then ramped to 280 °C at 20 °C/min and kept at 280 °C for 10 min.

The MS detector (quadrupole) was operated in SIM mode. The list of monitored ions for each of the analyzed compounds is shown in Tables S2–S5. Each sample was injected and analyzed four times with GC/MS—each time using a different SIM program for different analytes to have up to 10 ions in the ion group (2–3 ions per compound).

### 3.4. Data Processing

A virtual machine with eight processors (Intel Core i7 9xx, Intel Co., Santa Clara, CA, USA) and 16 GB RAM was used for Windows-based applications. All software was operated with 64-bit Windows 8.1 Enterprise operating system (Microsoft Co., Redmont, WA, USA). MassHunter Workstation Software Quantitative Analysis ver. B.08.00 (Agilent Technologies Inc., Santa Clara, CA, USA), MS Excel 2016 (Microsoft Co., Redmont, WA, USA) and RKWard version 0.7.0b were used for the chromatogram postprocessing and calculations.

## 4. Conclusions

Evaporation of dichloromethane in a stream of nitrogen causes a loss of analytes proportional to the volatility of the substance. When heating the sample (at 40 °C), this loss can be significant (even over 70–80%). Evaporation without heating (lasting longer) improved recovery for PCBs and OCPs (average recoveries greater than or equal to 90%) and could be an alternative way of running the process (no keeper needed). However, in the case of OPPs and o-hydroxybiphenyl, evaporation under such conditions cannot be used due to the adsorption of the analytes on the vial surface.

When evaporating while heating at 40 °C, the use of any keeper usually yielded higher recoveries for all analytes than when evaporation was carried out without it. For PCBs, the best keeper was 1-octanol (with recovery close to 100%), but for the rest of the keepers tested, the average recovery was also quite high (above 80%). Similar results were obtained for OCPs, with the exception of toluene where the average recovery was lower (about 70%). The same keeper (1-octanol) can also be used for pyrimethanil analysis. In the case of PAHs and o-hydroxybiphenyl, isooctane was the most suitable keeper (the highest recoveries). Particular care should be taken when choosing a less volatile solvent as a keeper (1-octanol and dodecane in these experiments), as the reverse solvent effect may occur with more volatile compounds (such as lower mass PAHs and o-hydroxybiphenyl). For OPPs, three keepers are suitable for the evaporation of DCM: 1-octane, nonane and isooctane (recoveries of above 96%).

It should be noted that recovery depends on many variables, such as the initial volume and type of solvent to be evaporated, the conditions of the evaporation process (temperature, gas flow) and the concentration and type of analytes dissolved in the solvent, but the results in this work give the general direction of the influence of the tested parameters on the process. They can be useful in selecting an appropriate keeper for a specific group of analytes and finding an alternative keeper to those commonly used, especially in the case of recovery problems.

## Figures and Tables

**Table 1:** Recoveries and RSDs (%) achieved in the polychlorinated biphenyl (PCB) evaporation experiments.

Experiment No.	1	2	3	4	5	6	7
Experiment or Keeper Name/Analyte	*w*/*o* Keeper	*w*/*o* Keeper, *w*/*o* Heating	Isooctane	Toluene	Nonane	1-Octanol	Dodecane
PCB 10	28.2	90.5	79.5	73.9	82.6	100.6	77.8
	(61.6)	(2.7)	(2.7)	(6.7)	(0.8)	(1.6)	(1.9)
PCB 28	45.3	94.2	83	83.5	86.3	106.6	82.2
	(12.8)	(3.0)	(2.4)	(5.0)	(1.3)	(2.3)	(2.0)
PCB 52	54.7	90.5	84.8	72.9	86.0	102.1	77.8
	(7.2)	(2.8)	(1.7)	(4.5)	(0.9)	(3.5)	(2.3)
PCB 153	71.5	89.3	85.7	81.8	85.8	92.4	80.4
	(1.6)	(1.3)	(2.0)	(2.1)	(0.5)	(1.4)	(3.7)
PCB 137	73.9	88.8	85.2	85.4	85.6	91.3	79.7
	(1.2)	(1.2)	(2.4)	(3.8)	(1.0)	(2.4)	(2.9)
PCB 180	81.0	89.2	87.2	91.6	87.3	92.4	82.6
	(1.2)	(3.0)	(2.1)	(2.3)	(0.6)	(1.7)	(3.3)
Average	59.1	90.4	84.2	81.5	85.6	97.6	80.1
	(14.3)	(2.3)	(2.2)	(4.1)	(0.9)	(2.1)	(2.7)
Number of cpds with recovery ∈ [90;110]	0	3	0	1	0	6	0

RSDs given in parentheses; *w*/*o*, without; cpds, compounds.

**Table 2:** Recoveries and RSDs (%) achieved in organochlorine pesticide (OCP) evaporation experiments.

Experiment No.	1	2	3	4	5	6	7
Experiment or Keeper Name/Analyte	*w*/*o* Keeper	*w*/*o* Keeper, *w*/*o* Heating	Isooctane	Toluene	Nonane	1-Octanol	Dodecane
α-HCH	34.3	89.8	78.6	60.9	87.3	98.9	77.4
	(32.9)	(11.9)	(1.6)	(6.3)	(2.0)	(2.7)	(3.5)
β-HCH	64.7	105.8	86.4	69.4	91.6	101.4	78.6
	(3.8)	(7.7)	(1.9)	(3.0)	(1.6)	(1.4)	(2.9)
Lindane	35.0	91.5	77.4	56.6	80.4	99.8	78.7
	(24.2)	(9.2)	(2.2)	(7.6)	(2.1)	(3.5)	(2.9)
δ-HCH	59.7	69.4	81.9	65.7	87.0	104.4	79.2
	(5.1)	(10.3)	(2.3)	(6.5)	(3.4)	(2.0)	(1.7)
Heptachlor	46.9	81.6	82.3	60.4	85.8	92.2	78.3
	(16.1)	(1.8)	(3.0)	(8.0)	(2.4)	(9.0)	(1.5)
Aldrin	45.0	64.2	79.1	58.5	84.3	93.3	72.5
	(16.5)	(7.6)	(2.1)	(8.2)	(1.3)	(15.2)	(5.0)
Heptachlor epoxide	58.9	88.2	85.5	66.2	89.5	89.2	74.1
	(4.5)	(1.4)	(2.1)	(6.3)	(1.0)	(11.7)	(5.1)
α-endosulfan	64.3	98.6	84.6	68.0	88.1	93.5	89.3
	(4.5)	(5.1)	(2.6)	(5.2)	(2.4)	(12.4)	(4.7)
4,4′-DDE	66.5	92.5	85.5	72.9	89.1	93.7	76.3
	(1.4)	(6.8)	(1.7)	(3.7)	(2.4)	(3.5)	(3.5)
Dieldrin	62.9	98.8	81.6	70.3	86.3	91.5	74.6
	(2.2)	(13.9)	(2.0)	(3.7)	(1.5)	(4.6)	(5.6)
4,4’-DDD	82.8	93.0	91.3	83.4	94.3	93.8	84.2
	(1.1)	(2.8)	(1.2)	(1.9)	(0.7)	(4.4)	(4.8)
4,4’-DDT	72.7	104.7	80.4	79.0	78.1	88.1	86.3
	(6.3)	(1.3)	(4.9)	(1.3)	(5.0)	(7.3)	(3.9)
Endosulfan sulfate	85.0	92.4	89.6	88.0	87.4	95.0	77.8
	(1.1)	(1.3)	(2.5)	(1.4)	(0.9)	(2.9)	(5.1)
Average	59.9	90.0	83.4	69.2	86.9	95.0	79.0
	(9.2)	(6.2)	(2.3)	(4.9)	(2.0)	(6.2)	(3.9)
Number of cpds with recovery ∈ [90;110]	0	8	1	0	2	11	0

RSDs given in parentheses; *w*/*o*, without; cpds, compounds.

**Table 3:** Recoveries and RSDs (%) achieved in the polycyclic aromatic hydrocarbon (PAH) evaporation experiments.

Experiment No.	1	2	3	4	5	6	7
Experiment or Keeper Name/Analyte	*w*/*o* Keeper	*w*/*o* Keeper, *w*/*o* Heating	Isooctane	Toluene	Nonane	1-Octanol	Dodecane
Naphthalene	20.6	53.9	96.2	33.9	77.7	–	–
	(7.3)	(13.8)	(4.1)	(1.6)	(8.5)		
2-Methylnaphthalene	22.3	89.4	97.3	48.4	96.0	–	62.4
	(25.6)	(4.7)	(11.3)	(2.5)	(12.5)		(2.3)
1-Methylnaphthalene	22.0	49.0	96.9	54.3	81.8	–	65.5
	(24.5)	(4.8)	(6.0)	(4.3)	(7.4)		(4.4)
Acenaphthylene	31.3	29.1	97.5	57.1	82.0	–	38.5
	(30.0)	(9.0)	(4.9)	(4.7)	(3.2)		(26.1)
Acenaphthene	32.5	69.3	102.3	54.5	75.3	–	48.2
	(39.3)	(4.7)	(4.5)	(7.1)	(3.9)		(3.9)
Fluorene	34.4	79.1	98.4	61.6	81.0	91.9	76.2
	(36.3)	(4.4)	(8.2)	(3.5)	(3.1)	(2.7)	(2.3)
Phenanthrene	49.5	59.2	96.7	63.6	93.5	99.3	84.0
	(23.3)	(4.8)	(7.1)	(2.1)	(3.3)	(4.5)	(2.4)
Anthracene	41.6	59.2	83.4	53.9	89.7	86.0	45.1
	(23.0)	(4.8)	(3.3)	(5.6)	(6.2)	(5.3)	(2.7)
Fluoranthene	86.9	88.4	100.2	79.5	81.2	91.0	86.0
	(5.5)	(2.8)	(7.4)	(4.4)	(2.1)	(3.3)	(1.7)
Pyrene	83.1	67.2	98.4	77.1	78.0	101.1	80.8
	(3.5)	(5.5)	(2.6)	(2.4)	(2.8)	(5.0)	(1.8)
Benz[a]anthracene + Chrysene	97.5	81.3	92.1	97.9	94.1	91.5	77.3
	(2.6)	(3.1)	(8.4)	(6.3)	(6.2)	(1.1)	(1.1)
Benzo[b]fluoranthene	104.2	62.0	85.8	100.0	89.6	93.5	101.2
	(3.3)	(11.4)	(8.7)	(5.8)	(5.5)	(3.4)	(3.2)
Benzo[k]fluoranthene	100.3	52.1	91.4	93.0	90.3	91.0	101.2
	(3.3)	(10.2)	(6.6)	(0.9)	(3.4)	(3.2)	(3.2)
Benzo[a]pyrene	83.1	–	89.1	79.2	88.4	86.9	105.5
	(9.3)	–	(7.4)	(6.4)	(4.3)	(13.1)	(6.8)
Indeno[1,2,3-cd]pyrene + Dibenz[a,h]anthracene	101.4	44.5	99.2	106.8	98.0	93.4	109.8
	(2.6)	(23.4)	(5.5)	(9.3)	(11.9)	(7.5)	(7.5)
Benzo[ghi]perylene	102.7	32.0	103.1	107.1	98.7	85.4	114.5
	(4.6)	(11.2)	(8.4)	(4.1)	(6.9)	(6.6)	(9.3)
Average	63.3	61.1	95.5	73.0	87.2	91.9	79.8
	(15.3)	(7.9)	(6.5)	(4.4)	(5.7)	(5.1)	(5.2)
Number of cpds with recovery ∈ [90;110]	5	0	13	5	6	8	4

RSDs given in parentheses; *w*/*o*, without; cpds, compounds.

**Table 4:** Recoveries and RSDs (%) achieved in the organophosphorus pesticide (OPP) evaporation experiments.

Experiment No.	1	2	3	4	5	6	7
Experiment or Keeper Name/Analyte	*w*/*o* Keeper	*w*/*o* Keeper, *w*/*o* Heating	Isooctane	Toluene	Nonane	1-Octanol	Dodecane
Ethoprophos	70.3	n.d.	106.5	88.4	102.8	101.6	83.8
	(9.6)	–	(1.5)	(4.9)	(1.3)	(5.0)	(3.2)
Fenchlorphos	61.0	n.d.	87.9	68.7	92.5	100.0	83.2
	(5.0)	–	(2.1)	(4.6)	(1.3)	(3.8)	(3.6)
Chlorpyrifos	66.4	n.d.	92.4	74.6	94.7	98.6	82.0
	(1.2)	–	(2.3)	(2.9)	(1.1)	(1.6)	(3.3)
Prothiofos	77.6	n.d.	98.3	81.9	97.3	98.6	85.5
	(1.4)	–	(2.3)	(1.2)	(0.9)	(1.5)	(2.4)
Average	68.8	–	96.3	78.4	96.8	99.7	83.6
	(4.3)	–	(2.0)	(3.4)	(1.1)	(3.0)	(3.1)
Number of cpds with recovery ∈ [90;110]	0	0	3	0	4	4	0

(RSDs given in parentheses; *w*/*o*, without; cpds, compounds).

**Table 5:** Recoveries and RSDs (%) achieved in the o-hydroxybiphenyl and pyrimethanil evaporation experiments.

Experiment No.	1	2	3	4	5	6	7
Experiment or Keeper Name/Analyte	*w*/*o* Keeper	*w*/*o* Keeper, *w*/*o* Heating	Isooctane	Toluene	Nonane	1-Octanol	Dodecane
o-Hydroxybiphenyl	43	n.d.	96.6	62.8	95.8	n.d.	n.d.
	(37.6)	–	(3.1)	(9.4)	(7.8)	–	–
Pyrimethanil	78.4	76.4	58.2	70.5	59.8	107.4	51.9
	(1.4)	(6.0)	(6.6)	(6.7)	(2.9)	(8.3)	(12.4)

(RSDs given in parentheses; *w*/*o*, without).

## Supplementary Materials

Table S1: Physical properties of dichloromethane (solvent) and keepers, Table S2: Retention time, monitored ions, MS SIM group and physical properties of PCBs, Table S3: Retention time, monitored ions, MS SIM group and physical properties of OCPs, Table S4: Retention time, monitored ions, MS SIM group and physical properties of PAHs, Table S5: Retention time, monitored ions, MS SIM group and physical properties of OPPs, o-hydroxybiphenyl and pyrimethanil, Figure S1: Diagram of a homemade nitrogen evaporator.
