# Supplementary material for: Evaluation of Solvents Used as Keepers in the Determination of Organic Pollutants by GC/MS
Source: Molecules. 2020 Sep 25;25(19):4419. doi: 10.3390/molecules25194419 (PMC7582485; doi:10.3390/molecules25194419)
Supplement: Supplementary file 1 [file molecules-25-04419-s001.pdf]

Supplementary materials

Evaluation of solvents used as keepers in the determination of organic pollutants by GC/MS

Łukasz Dąbrowski

UTP University of Science and Technology, Faculty of Chemical Technology and Engineering, Department of Food Analysis and Environmental Protection, 3 Seminaryjna Street, 85-326 Bydgoszcz, POLAND, phone: +48 523749014, fax: +48 523749005, e-mail: lukas@utp.edu.pl

The physical properties of the compounds were taken from the EPI Suite computer database and estimation program [1]. The following abbreviations are used in the tables: MW – average molecular weight, BP – boiling point, VP – vapour pressure, Q1, Q2: qualifier ions.

Table S1. Physical properties of dichloromethane (solvent) and keepers.

compound	BP [°C]	VP [mm Hg]	LogP
dichloromethane	39.7	435	1.25
isooctane	99.1	49.3	4.09
toluene	110.5	28.4	2.73
nonane	150.7	4.45	5.65
1-octanol	195.1	0.0794	3.00
dodecane	216.2	0.135	6.10

values underlined: calculated with EPI Suite 4.11

Table S2. Retention time, monitored ions, MS SIM group, and physical properties of PCBs.

compound	RT [min]	SIM group	monitored ions m/z target, Q1, Q2 [amu]	MW [g/mol]	BP [°C]	VP [mm Hg]	LogP
PCB 10	7.19	1	222, 152, 224	222	320	0.000191	4.98
PCB 28	8.38	2	256, 258, 186	258	341	0.000195	5.62
PCB 52	8.68	2	292, 290, 220	292	360	8.45E-06	6.09
PCB 153	10.25	3	360, 362, 290	361	397	3.75E-06	6.34
PCB 137	10.53	3	360, 362, 290	361	397	5.81E-07	7.44
TPH (IS)	10.65	3	326, 325, 215	326	441	6.28E-06	4.59
PCB 180	11.24	4	394, 396, 324	395	416	1.30E-07	8.27

values underlined: calculated with EPI Suite 4.11

Table S3. Retention time, monitored ions, MS SIM group, and physical properties of OCPs.

compound	RT [min]	SIM group	monitored ions m/z target, Q1, Q2 [amu]	MW [g/mol]	BP [°C]	VP [mm Hg]	LogP
◎-HCH	7.58	1	181, 183, 219	291	288	4.50E-05	3.80
◎-HCH	7.81	1	181, 183, 219	291	304	3.60E-07	3.78
lindane	7.88	1	181, 183, 219	291	323	3.20E-05	3.72
◎-HCH	8.09	1	181, 183, 219	291	304	3.52E-05	4.14
heptachlor	8.54	2	272, 274, 100	373	310	4.00E-04	6.10
aldrin	8.88	3	263, 265, 261	365	330	1.20E-04	6.50
heptachlor epoxide	9.24	4	353, 355, 351	389	341	1.95E-05	4.98
◎-endosulfan	9.58	5	241, 195, 263	407	401	1.73E-07	3.83
4,4′-DDE	9.73	5	246, 248, 318	318	336	6.00E-06	6.51
dieldrin	9.82	5	263, 279, 261	381	330	5.89E-06	5.40
endrin	10.03	5	263, 281, 345	381	340	3.00E-06	5.20
4,4'-DDD	10.12	6	235, 237, 165	320	350	1.35E-06	6.02
endrin aldehyde	10.28	6	345, 281, 250	381	340	1.70E-05	4.80
4,4'-DDT	10.47	7	235, 237, 165	354	260	1.60E-07	6.91
endosulfan sulfate	10.51	7	387, 272, 274	423	410	2.80E-07	3.66
TPH (IS)	10.65	7	326, 325, 215	326	441	6.28E-06	4.59
methoxychlor	11.04	8	227, 227, –	346	346	2.03E-07	5.08

values underlined: calculated with EPI Suite 4.11

Table S4. Retention time, monitored ions, MS SIM group, and physical properties of PAHs

Compound	RT [min]	SIM group	Monitored Ions m/z target, Q1, Q2 [amu]	MW [g/mol]	BP [°C]	VP [mm Hg]	LogP
naphthalene	5.29	1	128, –, –	128	218	0.085	3.30
2-methylnaphthalene	5.79	2	142, 141, 115	142	241	0.055	3.86
1-methylnaphthalene	5.85	2	142, 141, 115	142	245	0.067	3.87
Acenaphthylene	6.47	3	152, 151, 153	152	280	0.00668	3.94
Acenaphthene	6.62	3	153, 152, 154	154	279	0.00215	3.92
Fluorene	7.09	4	166, 165, –	166	295	0.0006	4.18
Phenanthrene	8.02	5	178, 176, –	176	340	0.000121	4.46
Anthracene	8.07	5	178, 176, –	178	340	6.53E-06	4.45
Fluoranthene	9.32	6	202, 200, –	202	384	9.22E-06	5.16
Pyrene	9.58	6	202, 200, –	202	404	4.50E-06	4.88
TPH (IS)	10.65	7	326, 325, 215	326	<u>441</u>	<u>6.28E-06</u>	4.59
Benz[a]anthracene*	11.09	8	228, 226, —	228	438	2.10E-07	5.76
Chrysene*	11.15	8	228, 226, —	227	448	6.23E-09	5.81
Benzo[b]fluoranthene	13.11	9	252, 250, —	252	<u>443</u>	5.00E-07	5.78
Benzo[k]fluoranthene	13.15	9	252, 250, —	252	480	9.65E-10	6.11
Benzo[a]pyrene	13.91	9	252, 250, —	252	495	5.49E-09	6.13
Indeno[1,2,3-cd]pyrene*	17.68	10	276, 277, 278	276	536	<u>1.25E-10</u>	<u>6.70</u>
Dibenz[a,h]anthracene*	17.88	10	276, 277, 278	278	524	9.55E-10	6.75
Benzo[ghi]perylene	18.72	10	276, 277, 278	276	>500	1.00E-10	6.63

**compounds quantified as a sum; values underlined: calculated with EPI Suite 4.11*

Table S5. Retention time, monitored ions, MS SIM group, and physical properties of OPPs, o-hydroxybiphenyl and pyrimethanil

Compound	RT [min]	SIM group	Monitored Ions m/z target, Q1, Q2 [amu]	MW [g/mol]	BP [°C]	VP [mm Hg]	LogP
Ethoprophos	7.19	1	158, 200, 242	242	<u>322</u>	0.00038	3.59
Fenchlorphos	8.56	3	285, 287, 125	322	<u>349</u>	7.50E-05	4.88
Chlorpyrifos	8.85	4	314, 316, 199	351	<u>377</u>	2.03E-05	4.96
Prothiofos	9.67	5	309, 267, 162	345	<u>386</u>	<u>9.40E-06</u>	5.67
TPH (IS)	10.65	6	326, 325, 215	326	<u>441</u>	<u>6.28E-06</u>	4.59
o-hydroxybiphenyl	6.79	1	170, 169, 141	170	286	0.002	3.09
pyrimethanil	7.97	2	198, 199, 200	199	<u>325</u>	1.65E-05	2.84

values underlined: calculated with EPI Suite 4.11

1. US EPA. [2012]. Estimation Programs Interface Suite™ for Microsoft® Windows, v 4.11. United States Environmental Protection Agency, Washington, DC, USA.

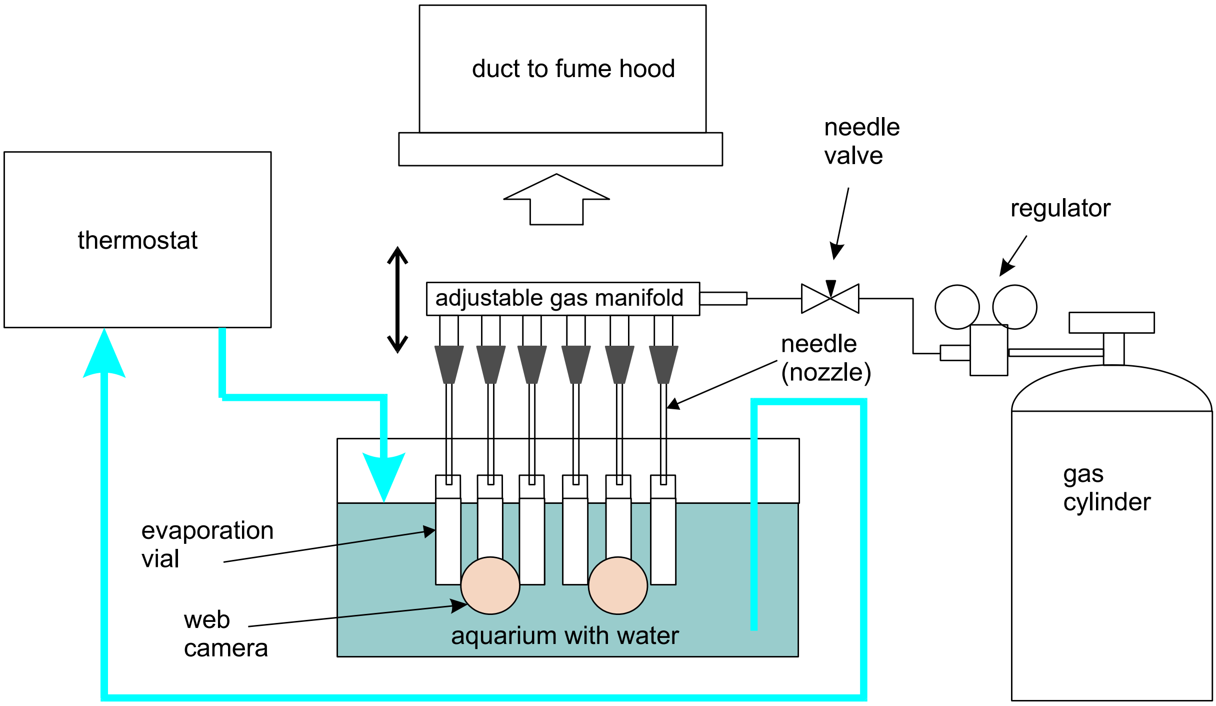

Figure S1. Diagram of a homemade nitrogen evaporator
